# The Origin of Moles

**Published:** 1853

**Authors:** Thomas Lipscomb

**Affiliations:** Shelbyville, Tenn.


ARTICLE V.—*The Origin of Moles.*—By THOMAS LIPSCOMB M. D. of Shelbyville, Tenn., in a letter to Prof. Yandell.

*Dear Sir:* I indulged the hope that a paper which I furnished for the April number of *The Western Journal of Medicine and Surgery*, on the origin of Moles, would have attracted the attention of others of more enlarged experience, to this subject, and elicited something either contradictory or corroborative of the theory advanced in the paper above referred to. Other subjects far less important in a practical point of view, have received the most patient investigation and elaborate research from numbers of the profession of no ordinary ability. When viewed in connection with the extreme liability to alarming hemorrhage consequent on the natural efforts for the expulsion of those fleshy masses, we can scarcely conceive of anything more vitally important; and should future observation confirm the correctness of the theory which I advanced, and females informed of the fearful risk they incur by continuing to nurse their children after the re-establishment of the catamenial function with regularity, after a cessation of that function for ten, twelve, or thirteen months, many threatening scenes would be prevented, as well as many valuable lives thereby saved.

Since the publication of the paper above alluded to, I have met with but two cases of mole or germen falsum, one of them occurring early in 1851, and the other case the 27th of January last, the first a white, and the last a black woman. I learned by minute inquiry the circumstances of each case. In the first case the catamenia failed to appear for sixteen months after the last confinement, then appeared with regularity, for several periods during which time lactation was continued. The catamenia were now interrupted for three periods, when the lady was attacked with uterine hemorrhage. When I was called to her, and upon making a vaginal and uterine exploration, I found it necessary to aid the uterus in expelling its contents, which proved to be a firmly-organized mass weighing about one ounce and a half. The next conception of this lady (as she had discontinued nursing), was a healthy one, as was proved by her giving birth to a healthy child about eleven months afterwards. The historical circumstances of the black woman's case were so entirely similar that a detail of them is wholly useless. Of course we cannot speak of the results that may follow in her case, the delivery of the mole having so recently occurred.

Respectfully, THOS. LIPSCOMB.

Feb. 4th, 1854.

